# Identification of a Novel α-herpesvirus Associated with Ulcerative Stomatitis in Donkeys 

**DOI:** 10.3201/eid2612.200201

**Published:** 2020-12

**Authors:** Vito Martella, Gianvito Lanave, Michele Camero, Vittorio Larocca, Eleonora Lorusso, Cristiana Catella, Paolo Capozza, Maria Tempesta, Canio Buonavoglia

**Affiliations:** University of Bari, Valenzano, Italy

**Keywords:** donkeys, EHV, equids, Equus asinus, glycoproteins, herpesviruses, mammillitis, phylogenic analysis, stomatitis, varicellovirus, Varicellovirus, vesiculoviruses, zoonoses

## Abstract

An outbreak of ulcerative stomatitis was observed in a donkey (*Equus asinus*) dairy herd. Similar lesions were also observed on the dams’ udders and, sporadically, in genital areas. The lesions typically resolved in 1–3 weeks. An α-herpesvirus, *Varicellovirus*, genetically related to equid herpesvirus type 3, was identified.

Vesicular stomatitis is a consequential disease of equids. Vesicular stomatitis virus (family *Rhabdoviridae*, genus *Vesiculovirus*) is a major infectious agent with zoonotic potential that is common in the Americas. A few other infectious viral agents (equine arteritis virus, bunyavirus, caliciviruses, equine adenoviruses, and herpesviruses) have been associated with this condition in horses, but on several occasions the etiology of vesicular and ulcerative stomatitis remains undiagnosed (*1*). Noninfectious etiology may include plant- and drug-related toxicoses and photosensitization (*1*). 

In October 2011, an outbreak of ulcerative stomatitis started in a donkey (*Equus asinus*) dairy herd, comprising 106 animals, located in the prefecture of Bari, Apulia region, Italy. The outbreak appeared related to the introduction of a pregnant female 8 weeks before the onset of the disease. The mare was clinically healthy and in good physical condition at arrival and gave birth to a foal after 7 weeks. Clinical signs developed in neither the dam nor the foal. 

Initially, the outbreak affected 34 animals (17 lactating mares with their foals). This group was separated with wood fences from the other animals, but not strictly. Fever and small nodular lesions, evolving into painful ulcers, were observed on the oral mucosa, tongue, and skin around the lips of young animals (2 weeks–4 months old) (Figure 1). Similar lesions were also observed sporadically on the dams’ udders and genital areas. The lesions typically resolved in 1–3 weeks. The herd owner reported weight loss in foals and interruption of lactation in dams. Two weeks after onset in the original group, the disease was observed in a separate group of animals, comprising 63 adult or yearling females and 5 yearling males. In this group, however, oral lesions were observed only in 5 yearlings and 1 mare. A third group of 4 adult males was kept apart from the other animals and was not affected by the disease. 

**Figure 1:**
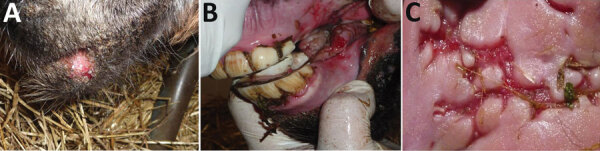
Foals with herpesviral infection, part of an outbreak of ulcerative stomatitis in a donkey dairy herd, Bari, Italy. Ulcerative lesions were observed on the lips (A), oral mucosa (B), and tongue (C).

Oral swab and serum samples collected from 8 animals with acute disease were sent to the laboratories at the University of Bari (Valenzano, Italy) for virologic investigation. Using an electron microscope, we observed herpesvirus-like particles in the oral swabs and detected herpesvirus DNA using consensus herpesvirus primers for the DNA polymerase and inverse terminase (*2*,*3*). We used BLASTn (https://blast.ncbi.nlm.nih.gov) to search the GenBank genetic sequence database and found the virus to be highly related to equid herpesvirus (EHV) 3 in the DNA polymerase (93.35% nt identity) and terminase (90.71% nt identity) regions. 

We isolated the virus onto equine dermal cells from oral swab specimens. The virus was titrated and used for screening serum samples collected from the donkeys in virus neutralization assays. We detected specific neutralizing antibodies in the serum samples collected from approximately three quarters (80/106) of the animals 2 months after the beginning of the outbreak but not in the serum samples of 8 animals with acute infection, suggesting seroconversion. 

To sequence the DNA of the isolate, we performed next generation sequencing using the Illumina MiSeq platform (https://www.illumina.com) and used Nextera XT (Illumina) for library preparation. We obtained the full genome sequence (147,607 bp) of asinine herpesvirus (AsHV) strain AsHV/Bari/2011/740 and annotated it using the software ORF Finder (https://www.bioinformatics.org). 

On full genome sequence analysis, strain AsHV/Bari/2011/740 appeared genetically related (87.02% nt identity) to EHV-3 strain AR/2007/C3A (accession no. KM051845) (subfamily *α-Herpesviridae*, genus *Varicellovirus*). Three different full-length gene targets (glycoproteins B, C, and D) of strain AsHV/Bari/2011/740 were aligned with cognate sequences representative of the genus *Varicellovirus* listed by the International Committee on Taxonomy of Viruses (https://talk.ictvonline.org) by using Geneious software version 9.1.8 (Biomatters Ltd., https://www.geneious.com) and the MAFFT algorithm (*4*). We performed phylogenetic analyses with MEGAX software (https://www.megasoftware.net) (*5*) using the maximum-likelihood method with the general time-reversible model, a proportion of invariant sites, and a discrete gamma distribution (5 categories) to model evolutionary rate differences among sites, and bootstrap analyses with 1,000 pseudoreplicate datasets. In the consensus phylogenetic trees (Figure 2), strain AsHV/Bari/2011/740 was closely related to EHV-3 sequences and distantly related to other members of the genus *Varicellovirus*. We deposited the full-genome sequence of strain AsHV/Bari/2011/740 in the GenBank database (accession no. MT012704). 

**Figure 2:**
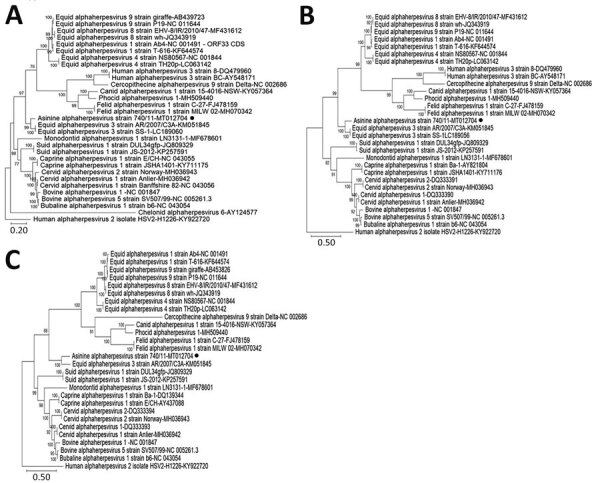
Phylogenetic trees based on the full-length nucleotide sequence of different target genes from the AsHV/Bari/2011/740 strain isolated from a donkey dairy herd, Bari, Italy (black dots), or retrieved from the International Committee on Taxonomy of Viruses database. A) Glycoprotein B; B) glycoprotein C; C) glycoprotein D. Posterior output of the tree was derived from maximum-likelihood inference using a general time-reversible model, a proportion of invariable sites, a gamma distribution of rate variation across sites, and a subsampling frequency of 1,000. Posterior probability values >70% are indicated on the tree nodes. Human alphaherpesvirus 2 isolate HSV-2-H1226 (genus *Simplesvirus*) strain (GenBank accession no. KY922720) was used as an outgroup. Scale bars indicate nucleotide substitutions per site. AsHV, asinine herpesvirus.

To date, several herpesviruses have been discovered in donkeys (Table). AsHV type 1, also called EHV-6, is an α-herpesvirus associated with ulcerative lesions (*6*). AsHV-2 (EHV-7) is a γ-herpesvirus identified in leukocytes of healthy animals (*7*). AsHV-3 (EHV-8) was isolated from the nose of immunodepressed animals (*8*) and was classified as an α-herpesvirus on the basis of the glycoprotein G sequence and poor antigenic cross-reactivity with EHV-1 and EHV-4 (*8*–*10*). AsHV-4, -5, and -6 are γ-herpesviruses identified from donkeys with interstitial pneumonitis (*2*,*11*). 

**Table:** List of equid herpesviruses recognized in the ICTV database or other sources*

Species	Subfamily	Related to	Other designations	Source
EHV-1	Alpha	NA	Equine abortion	ICTV
EHV-2	Gamma	NA	NA	ICTV
EHV-3	Alpha	NA	Coital exantema	ICTV
EHV-4	Alpha	NA	Equine rhinopneumonitis	ICTV
EHV-5	Gamma	NA	NA	ICTV
EHV-6	Alpha	EHV-3	AsHV-1	(*6*); this study
EHV-7	Gamma	EHV-2/EHV-5	AsHV-2	ICTV
EHV-8	Alpha	EHV-1	AsHV-3	ICTV
AsHV-4	Gamma	EHV-2/EHV-5	NA	(*11*)
AsHV-5	Gamma	EHV-2/EHV-5	NA	(*11*)
AsHV-6	Gamma	EHV-2/EHV-5	NA	(*2*)
EHV-9	Alpha	EHV-1	Zebra, gazelle, giraffe herpesviruses	ICTV
*AsHV, asinine herpesvirus; EHV, equine herpesvirus; ICTV, International Committee on Taxonomy of Viruses; NA, not applicable.				

We report the detection and isolation of a novel AsHV from an outbreak of vesicular and ulcerative stomatitis and mammillitis in a donkey dairy herd. By comparing it with other herpesvirus sequences from the databases, we identified 3 targets (glycoproteins B, C, and D) for which the sequences were available across all the varicelloviruses listed in the ICTV database and that were used for phylogenetic analysis. In these analyses, the AsHV strain appeared similar to EHV-3. By reviewing the literature, we found another donkey herpesvirus, AsHV-1, genetically related to EHV-3 on the basis of restriction enzyme and hybridization analyses (*6*). AsHV-1 was originally isolated from the vesicular and erosive lesions of the muzzle of a foal and the external genitalia and udder of its dam (*12*). Unfortunately, the prototype AsHV-1 is no longer available and it is not possible to determine its genome sequence for precise comparison (G.F. Browning, pers. comm.). It is possible that AsHV/Bari/2011 is actually an AsHV-1 strain, although this possibility cannot be confirmed.

Overall, based on the chronology of the health events observed in the herd, the tendency of herpesviruses to reactivate from latent infection under stress conditions, and the seroconversion observed in the monitored animals, we hypothesized that the newly introduced mare was the vehicle for herpesvirus infection in the herd, although this possibility could not be conclusively confirmed. Also, we screened only 8 animals during the acute phase of the disease, and we do not have an exact picture of the immunological status of the animals before the onset of the disease. 

In conclusion, we identified a novel AsHV, genetically related to EHV-3, from an outbreak of infectious ulcerative stomatitis in donkey foals. These findings extend the spectrum of pathologies potentially attributable to herpesviruses in donkeys. Considering the nature and shape of the lesions, the virus should be included in the differential diagnosis of vesicular and ulcerative stomatitis among equids. Also, it needs to be determined whether the novel AsHV can be transmitted to horses. 
